# LIVE AND DIE WITH DIGNITY: ALASKA NATIVE PERSPECTIVES AND EXPERIENCES ON DEMENTIA CAREGIVING AND FAMILY STIGMA

**DOI:** 10.1093/geroni/igae098.4303

**Published:** 2024-12-31

**Authors:** Steffi Kim, Jordan Lewis, Hannah Rebadulla, Lena Thompson, Murat Demir, Prince Iverson Alejo, Zoey Hilderbrand

**Affiliations:** University of Alaska, Anchorage, Alaska, United States; University of Minnesota Medical School, Duluth, Duluth, Minnesota, United States; University of Alaska Anchorage, Anchorage, Alaska, United States; University of Alaska Fairbanks, Fairbanks, Alaska, United States; University of Alaska Anchorage, Anchorage, Alaska, United States; University of Alaska, Anchorage, Alaska, United States; University of Anchorage Alaska, Anchorage, Alaska, United States

## Abstract

Family stigma (FS), referring to experiences of stigma by association, is the consequence of stigmatizing beliefs and attitudes. This study assessed FS among Alaska Native (AN) Alzheimer’s disease and related dementias (ADRD) caregivers spanning three domains: caregiver, layperson, and structural stigma. Stigma can prevent caregivers from seeking a diagnosis, treatment, or support services. Caregivers report limited knowledge of the disease, little engagement with support and resources, and social isolation. This pilot study used a mixed methods approach to outline ADRD-related stigma and its impact AN caregivers’ quality of life. Semi-structured 60-90-minute interviews guided by the adapted Family Stigma – Alzheimer’s Disease scale were conducted with thirteen female AN ADRD caregivers across Alaska. The caregiver’s mean age was 62, with an average of seven years of caregiving. Descriptive statistics were used to report the quantity and frequency of reported experiences of stigma. Qualitative inquiries allowed for the exploration of in-depth cultural context. Interviews were analyzed using deductive and inductive content analysis. Caregivers identified several sources of FS. Layperson and structural stigma items were endorsed most often. Caregiver stigma detailed that limited support results in high levels of distress and experiences of depression. Cultural values such as caring for the Elders were protective and led to family and community involvement in giving care. Stigma reduced AN ADRD caregiver’s quality of life in the areas of family cohesion, limited participation in social events, and impacted professional engagement negatively. This study can inform future work to decrease the stigma associated with AN ADRD.

